# Analyzing Large-Scale Studies: Benefits and Challenges

**DOI:** 10.3389/fpsyg.2020.577410

**Published:** 2020-12-09

**Authors:** Bernhard Ertl, Florian G. Hartmann, Jörg-Henrik Heine

**Affiliations:** ^1^Department of Human Sciences, Learning and Teaching With Media, Institute for Education, Universität der Bundeswehr München, Neubiberg, Germany; ^2^Department of Human Sciences, Methodology in the Social Sciences, Institute for Education, Universität der Bundeswehr München, Neubiberg, Germany; ^3^Center for International Student Assessment, TUM School of Education, Technical University of Munich, Munich, Germany

**Keywords:** large-scale assessments, ILSA, PISA, PIAAC, NEPS, educational psychology, learning and teaching

## Introduction

The analysis of (inter)national large-scale assessments (LSAs) promises representativity of their results and statistical power and has the ability to reveal even minor effects. LSAs' international grounding verifies previous findings that might previously have been biased by their focus on Western and industrialized countries. This contribution will discuss these promises, contextualizing them via methodical challenges and interpretation caveats that are able to tap the potential of LSAs for educational psychology. Evidence of this contribution is grounded in previous analyses of Program for International Student Assessment (PISA; Schleicher, 2019) and Program for the International Assessment of Adult Competencies (PIAAC; OECD, 2013), two internationally repeated cross-sectional studies. Many aspects we bring up can also apply to several other international large-scale studies, such as TIMSS, PIRLS, and ICILS.^1^ We also refer to the national longitudinal study German National Educational Panel Study (NEPS; Blossfeld et al., 2011) to include a perspective on longitudinal studies in this paper. Implications for large-scale studies within the context of learning and teaching round off our paper in its closing section.

## Promises

### Representativity and Impact

LSAs aim to survey representative (sub)samples of defined populations (e.g., OECD, 2013, section Caveats). This representativity can help them be more informative and provide stronger evidence for policymaking than traditional educational or psychological studies that often rely on convenience samples. Wagemaker (2014) discusses changes in educational policies as one of LSAs' impacts. Fischman et al. (2019) looked deeper inside the issue of LSAs' direct impact on educational policy, finding that several countries worldwide have established PISA-based educational goals (p. 12). They further report that LSA results are often used as triggers or levers for educational reforms, while also showing that several stakeholders mentioned that these kinds of studies actually hinder reforms when their focus is too much on simply reaching the stated indicators (see Rutkowski and Rutkowski, 2018).

### Longitudinal Perspective

A second LSA benefit is their long-time perspective. They either have been repeated cross-sectionally in several cycles (e.g., the PISA study takes place every 3 years; Schleicher, 2019) or show a longitudinal panel design, such as with NEPS that recently surveyed six starting cohorts in Germany over the past 10 years (Blossfeld and Roßbach, 2019). While the trend-study approach of PISA allows a measurement of how changes in educational policy or society may impact a defined sample (e.g., 15-year-old students in PISA; Schleicher, 2019), the longitudinal approach of NEPS enables background variables to be revealed, shedding light on how an individual's characteristics affect educational trajectories (Blossfeld and Roßbach, 2019). These procedures can be especially informative if a study like NEPS follows several cohorts that overlap at a certain point in time.

### Standardization

Besides representativity and the longitudinal perspective, LSAs provide standardized procedures, instruments, item pools, and test booklets (e.g., OECD, 2013). These standardizations ensure a survey setting and data that allow international comparisons (PIAAC and PISA) as well as comparisons between survey cycles (PIAAC and PISA) or waves (NEPS). An essential prerequisite for supporting these comparisons is the international cooperation for developing competency and performance measures as well as questionnaires (see, e.g., OECD, 2013). Furthermore, the standardized coding of survey data allows a certain level of matching to contextual and/or official data, e.g., labor market data, national examination statistics, or even geodata from microcom in NEPS (Schönberger and Koberg, 2018).^2^

### Statistical Power

Finally, the large sample sizes with LSAs provide a statistical power for analyses that allows detection on the individual level of even small effects, even if subsamples of the original population are analyzed. This helps to reveal effects that would have been overlooked in traditional educational or psychological studies. However, statistical power here decreases when analyses go beyond the individual level and focus on class, school, or national realms.

## Challenges

### Complexity of Analysis

These promises go along with analysis and interpretation challenges. The advantage of representativity in the context of economic sample sizes requires a complex weighting of each case. Consequently, all further analyses must include weights to be able to maintain representativity during analyses. Using stratification variables for sampling that differ across the participating countries to reflect different (educational) structures in their population requires complex variance estimation procedures. This is typically based on replicated estimation or bootstrap procedures (Rust, 1985; Lin et al., 2013) to prove significance statements. In addition, the principle of item sampling (e.g., Lord, 1965) typically used in competence assessment (see Rutkowski et al., 2013) results in design-related missing data points (see below), which are compensated by the plausible value (PV) techniques (e.g., von Davier et al., 2009; von Davier, 2013, and Marsman et al., 2016). Here, analysis procedures have to take not only one but also multiple (e.g., five, ten, or even more) variables (PV) as competence measures into account. However, these kinds of procedures are rare with traditional statistics programs,^3^ meaning representative analyses need either add-ons such as the IDB Analyzer^4^ or specifically developed packages for R (e.g., survey; BIFIEsurvey, or intsvy; see Heine and Reiss, 2019).

### Test Time

Another aspect relates to the extent of the questionnaires. People being surveyed can offer only a limited amount of time. This is typically compensated for in LSAs via two alternative approaches. A pragmatic and easily implemented approach is to apply very short scales for measuring traits and competencies. The NEPS panel, for example, measures the Big Five^5^ personality domains with only two items per dimension and vocational interests (the Big Six) with three items per dimension (see Wohlkinger et al., 2011). The issue of expectably low reliabilities and the respective validity is increasingly being discussed in psychological research (Rammstedt and Beierlein, 2014). A more demanding approach in terms of both implementation and later analysis is to use rotated booklet designs (e.g., Frey et al., 2009 and Heine et al., 2016). For computer-based assessments, adaptive test scenarios can usually further reduce the number of items (e.g., Kubinger, 2017). In both test designs, the items are appropriately distributed across different test booklets or even test scenarios. Test takers here often do not answer every item, which inevitably results in missing data points. With a suitable test design, this loss of data is typically completely random, although it still might require the use of data imputation methods which can be complicated to apply.^6^

### Missing Data and Imputation

Correspondingly, for the construction of short scales or *within-scale*^7^ booklet designs, LSAs often require general design decisions for the assessment of competencies. The NEPS data set for instance surveyed competencies for only about a third of the student cohort (FDZ-LIfBi, 2018), while PIAAC assessed the competency of problem solving in technology-rich environments just for parts of the sample (OECD, 2013) with the booklet designs described above. This means that there is no discrete competency value for an individual; the estimate for competency is based on PVs (e.g., von Davier et al., 2009), which are based on the theory of data imputation (see Rubin, 1987). Modeling longitudinal effects, e.g., by structural equation modeling, furthermore requires the availability of the target variables at specific waves in order to construct valid models.

### Invariance of Measurement

A recent OECD conference related to cross-country comparability of questionnaire scales (see Avvisati et al., 2019) identified measurement invariance as a core challenge for LSAs in general and for PISA studies as well (Van de Vijver et al., 2019). Among other methodological topics, participants from different countries discussed typical forms of analysis for verification of measurement invariance. A classical approach for the verification of the measurement invariance uses multigroup confirmatory factor analysis (MGCFA). Based on this, a widely accepted taxonomy includes configurational, metric, scalar, and residual measurement invariance (e.g., Putnick and Bornstein, 2016). The MGCFA approach however also has critical aspects ranging from insufficient subgroup sizes (even for LSA data), reduced test strength, and unknown distribution properties of the test statistics—especially when global model validation tests are used to assess the relative model fit of varyingly nested MGCFA models for levels of measurement invariance. Moreover, MGCFA rests on the assumption of a continuous scale for both the latent variable of interest and the response scales of the manifest indicators. When these strong assumptions of interval scales can be seriously questioned, different models from the IRT domain can be used for ordinal scales or methodology for classification like (multigroup) latent class analysis (MG-LCA—Eid et al., 2003 and Eid, 2019) for nominal scales. Some recent approaches in the LSA framework are founded upon Bayesian IRT models (e.g., Fox, 2010) or IRT residual fit statistics (see, e.g., Buchholz and Hartig, 2017). To establish an invariant scale on the item level, there are in fact some promising approaches to automated item selection to determine a scale, which fulfill predefined target criteria such as invariance across subsamples and cultures (e.g., Schultze and Eid, 2018).

### Item Formats and Response Sets

Extreme and middle response endorsement, cheating, socially desirable responding, and flat-lined response behavior are phenomena closely related to the issue of invariant measurement (see Heine, 2020). A critical discussion is currently taking place regarding whether innovative item formats (Kyllonen, 2013) such as *forced choice* measures (e.g., Bürkner et al., 2019) or *anchoring vignettes* to adjust distorted responses (e.g., Stankov et al., 2018) might lead to improved measurement when compared to classical rating scales.

### Classification Issues and Different Standards

Standardization and international comparability require the classification of responses, e.g., of vocational aspirations, by standardized classification schemes such as the ISCO-08. However, standardization is always subject to national practice and legislation, and although these schemes are in fact well-defined, they usually do not unambiguously map in alignment with national peculiarities; i.e., they often are only able to partially map national differences. Nursing is widely discussed as a prototypical challenge when it comes to international classification issues (see, e.g., Baumann, 2013 and Palmer and Miles, 2019) because it is distinguished with respect to the educational path (vocational vs. university background) as well as in terms of the scope of medical treatment a nurse is allowed to perform (see, e.g., Currie and Carr-Hill, 2013 and Gunn et al., 2019).

## Caveats

### Significance Does Not Mean Big Effects

Along with these challenges, LSAs also provide some interpretation caveats. The high sample sizes of large-scale studies support big statistical power (on the level of the individual) as a result frequent significance levels of *p* < 0.001 (or lower). Although this is strong when it comes to detecting even marginal differences, it also allows marginal effect sizes (zero effects) to become significant. So merely showing the significance of differences is not sufficient (e.g., Cohen, 1994 and Hunter, 1997) when analyzing large-scale studies; it is necessary to additionally discuss effect sizes (e.g., Snyder and Lawson, 1993).

### Horse Race Communication

Countries and states participating in international large-scale studies differ in both their schooling systems and general societal aspects. Just one example of this involves socioeconomic background variables and basic political and social convictions. Different immigration policies in different countries (see, e.g., Entorf and Minoiu, 2005 and Hunger and Krannich, 2015) can lead to a different population composition in so-called “non-native speaker groups,” or groups of people with low socioeconomic status might in turn influence (bias) the outcomes of these studies in cross-country comparisons much more than the factor of different school systems. Many international large-scale studies have very complex designs and analyses, and as a result, local or national aspects might be the most illustrative ones to communicate, even if they are not the most relevant ones when considering other educational factors. This often leads to a horse race discussion focusing on the position rather than on the peculiarities of the respective systems. While Rutkowski and Rutkowski (2018) describe how to deal with these peculiarities, the NEPS data use agreement prohibits comparisons between the German federal states^8^ to avoid precisely these issues.

## Implications for Learning and Teaching

We have discussed the promises, challenges, and caveats of LSAs. Benefits such as representativity and the long-time perspective go along with challenges such as the complexity of analysis and limited information (e.g., information loss due to classification issues, missing values, constructs not covered, and panel loss) as well as with further caveats for interpretation. This reflects a general issue of these studies, i.e., that their result might have the power to influence educational policies (see Fischman et al., 2019) while at the same time displaying difficulties in being appropriately communicated to teachers, principals, and policymakers due to their complexity. This makes it essential to communicate and transfer LSA evidence into practice in a manner that this is appropriate and understandable for a non-scientific audience, without trivializing its results.

The international perspective of many large-scale studies allows the stereotypes and preconditions that national studies cannot overcome to be reflected upon (see also Else-Quest et al., 2010). These include for example stereotyped gender differences in mathematics and science that in the Western world often favor boys—while PISA results on the other hand have disclosed that several countries show scores favoring girls in mathematics and an almost even distribution in science scores (OECD, 2015, p. 28f.). The study design thereby allows an analysis of the extent to which phenomena develop over time and between different countries, which is an essential aspect for evaluating changes in really any educational system. Incidentally, education always targets the development of individuals. So longitudinal follow-up surveys and analyses of cohorts may increase the benefits of these studies as they relate to learning and teaching.

To sum up, (inter)national large-scale studies can provide several benefits for research on learning and teaching in how they achieve a solid data set for investigating relevant effects. However, the formal comparability of study scores does not exactly reflect actual differences between states or educational systems without considering background variables and national social and educational specifics. Although these studies may mitigate the methodical shortcomings of traditional studies, especially the focus on Western white populations, they at the same time may reveal methodical challenges.

## Author Contributions

All authors listed have made a substantial, direct and intellectual contribution to the work, and approved it for publication.

## Conflict of Interest

The authors declare that the research was conducted in the absence of any commercial or financial relationships that could be construed as a potential conflict of interest.

## Footnotes

^1^See, e.g., Lenkeit and Schwippert (2018), Gustafsson (2018), von Maurice et al. (2017), and Rutkowski et al. (2010) for an overview of international large-scale studies.

^2^Matching to contextual data is typically required to preserve the anonymity of individuals and schools. Here, different levels of anonymization, starting from a segment of households up to the municipality level, may be observable (see Schönberger and Koberg, 2018). This kind of matching is usually implemented by the provider of the data set and may require further data access restrictions, e.g., that access is granted only in rooms with specific security precautions. Microcom enrichment may be restricted in some countries and for some studies.

^3^Analyses would be supported by multilevel structural equation modeling, e.g., in MPLUS, if the correct weights are appropriately used and the plausible values are correctly applied. However, the usability of this modeling is dependent on the complexity of the data set and decreases dramatically when nested plausible values are used, for example.

^4^https://www.iea.nl/data-tools/tools

^5^The Big Five is a set of personality variables including the dimensions of openness, conscientiousness, extraversion, agreeableness, and neuroticism (see Goldberg, 1990 and McCrae and John, 1992).

^6^The use of rotated booklet designs and/or adaptive testing usually leads to the imputation of data by the provision of plausible values for estimating test results (see next section). This increases the complexity of analyses (as mentioned in the previous section).

^7^The *within-scale booklet design* is used to describe the phenomenon that all constructs or scales are represented in all booklets, albeit with different and a reduced number of items.

^8^https://www.neps-data.de/Portals/0/NEPS/Datenzentrum/Datenzugangswege/Vertraege/NEPS_DataUseAgreement_en.pdf

## References

[B1] Avvisati F. Le Donné N. Paccagnella M. (2019). A meeting report: cross-cultural comparability of questionnaire measures in large-scale international surveys. Meas. Instrum. Soc. Sci. 1:8 10.1186/s42409-019-0010-z

[B2] Baumann A. (2013). What's in a name? The importance of definition and comparable data. Int. Nurs. Rev. 60, 75–77. 10.1111/j.1466-7657.2012.01046.x 23406240

[B3] Blossfeld H. P. Roßbach H. G. (Eds.). (2019). Education as a Lifelong Process: The German National Educational Panel Study (NEPS), 2nd Edn Wiesbaden: SpringerVS 10.1007/978-3-658-23162-0

[B4] Blossfeld H. P. Roßbach H. G. von Maurice J. (2011). Education as a lifelong process: The German National Educational Panel Study (NEPS). Zeitschrift Erziehungswissenschaft Sonderheft. 14, 19–34. 10.1007/s11618-011-0179-2

[B5] Buchholz J. Hartig J. (2017). Comparing attitudes across groups: An IRT-based item-fit statistic for the analysis of measurement invariance. Appl. Psychol. Meas. 5, 1–10. 10.1177/0146621617748323 31019359 PMC6463271

[B6] Bürkner P. C. Schulte N. Holling H. (2019). On the statistical and practical limitations of thurstonian IRT models. Educ. Psychol. Meas. 79, 827–854. 10.1177/0013164419832063 31488915 PMC6713979

[B7] Cohen J. (1994). The earth is round (p < .05). Am. Psychol. 49, 997–1003. 10.1037/0003-066X.49.12.99728368177

[B8] Currie E. J. Carr-Hill R. A. (2013). What is a nurse? Is there an international consensus? Int. Nurs. Rev. 60, 67–74. 10.1111/j.1466-7657.2012.00997.x 23406239

[B9] Eid M. (2019). “Multigroup and multilevel latent class analysis,” in Invariance Analyses in Large-Scale Studies, ed F. J. van de Vijver (Paris: OECD Publishing), 70–90.

[B10] Eid M. Langeheine R. Diener E. (2003). Comparing typological structures across cultures by multigroup latent class analysis. J. Cross Cult. Psychol. 34, 195–210. 10.1177/0022022102250427

[B11] Else-Quest N. M. Hyde J. S. Linn M. C. (2010). Cross-national patterns of gender differences in mathematics: a meta-analysis. Psychol. Bull. 136, 103–127. 10.1037/a0018053 20063928

[B12] Entorf H. Minoiu N. (2005). What a difference immigration policy makes: a comparison of PISA scores in Europe and traditional countries of immigration. German Econ. Rev. 6, 355–376. 10.1111/j.1468-0475.2005.00137.x

[B13] FDZ-LIfBi (2018). Codebook. NEPS Starting Cohort 5—First-Year Students. From Higher Education to the Labor Market. Scientific Use File Version 11.0.0. Retrieved from https://www.neps-data.de/Portals/0/NEPS/Datenzentrum/Forschungsdaten/SC5/11-0-0/SC5_11-0-0_Codebook_en.pdf

[B14] Fischman G. E. Topper A. M. Silova I. Goebel J. Holloway J. L. (2019). Examining the influence of international large-scale assessments on national education policies. J. Educ. Policy 34, 470–499. 10.1080/02680939.2018.1460493

[B15] Fox J. (2010). Bayesian Item Response Modeling. New York, NY: Springer New York 10.1007/978-1-4419-0742-4

[B16] Frey A. Hartig J. Rupp A. A. (2009). An NCME instructional module on booklet designs in large-scale assessments of student achievement: theory and practice. Educ. Meas. 28, 39–53. 10.1111/j.1745-3992.2009.00154.x

[B17] Goldberg L. R. (1990). An alternative description of personality: the big-five factor structure. J. Pers. Soc. Psychol. 59, 1216–1229. 10.1037/0022-3514.59.6.1216 2283588

[B18] Gunn V. Muntaner C. Ng E. Villeneuve M. Gea-Sanchez M. Chung H. (2019). Gender equality policies, nursing professionalization, and the nursing workforce: a cross-sectional, time-series analysis of 22 countries, 2000–2015. Int. J. Nurs. Stud. 99:103388. 10.1016/j.ijnurstu.2019.103388 31493758

[B19] Gustafsson J. E. (2018). International large-scale assessments: current status and ways forward. Scand. J. Educ. Res. 62, 328–332. 10.1080/00313831.2018.1443573

[B20] Heine J. H. (2020). Untersuchungen zum Antwortverhalten und zu Modellen der Skalierung bei der Messung psychologischer Konstrukte. München; Neubiberg: Universität der Bundeswehr.

[B21] Heine J. H. Mang J. Borchert L. Gomolka J. Kröhne U. Goldhammer F. Sälzer C. (2016). “Kompetenzmessung in PISA 2015,” in PISA 2015: Eine Studie zwischen Kontinuität und Innovation, eds K. Reiss, C. Sälzer, A. Schiepe-Tiska, E. Klieme, and O. Köller (Münster: Waxmann), 383–430.

[B22] Heine J. H. Reiss K. (2019). “Pisa 2018 – die Methodologie,” in PISA 2018 Grundbildung im internationalen Vergleich, eds K. Reiss, M. Weis, E. Klieme, and O. Köller (Münster: Waxmann), 241–258.

[B23] Hunger U. Krannich S. (2015). Einwanderungsregelungen im Vergleich: was Deutschland von anderen Ländern lernen kann. Bonn: Friedrich-Ebert-Stiftung.

[B24] Hunter J. E. (1997). Needed: a ban on the significance test. Psychol. Sci. 8, 3–7. 10.1111/j.1467-9280.1997.tb00534.x

[B25] Kubinger K. D. (2017). “Adaptive testing,” in Principles and Methods of Test Construction: Standards and Recent Advances. Vol. 3, Psychological Assessment - Science and Practice, eds K. Schweizer and C. DiStefano (Göttingen: Hogrefe), 104–119.

[B26] Kyllonen P. and Bertling, J. (2013). “Innovative questionnaire assessment methods to increase cross-country comparability,” in Handbook of International Large-Scale Assessment: Background, Technical Issues, and Methods of Data Analysis, eds L. Rutkowski, L. M. von Davier and D. Rutkowski (Boca Raton: Chapman and Hall/CRC), 277–285.

[B27] Lenkeit J. Schwippert K. (2018). Doing research with international assessment studies: methodological and conceptual challenges and ways forward. Assess. Educ. 25, 1–4. 10.1080/0969594X.2017.1352137

[B28] Lin C. Devon W. Lu W. Rust K. Sitter R. R. (2013). Replication variance estimation in unequal probability sampling without replacement: One-stage and two-stage. Can. J. Stat. Revue Canad. Stat. 41, 696–716. 10.1002/cjs.11200

[B29] Lord F. M. (1965). Item sampling in test theory and in research design. ETS Res. Bull. Series 1965, i−39. 10.1002/j.2333-8504.1965.tb00968.x

[B30] Marsman M. Maris G. Bechger T. Glas C. (2016). What can we learn from plausible values? Psychometrika 81, 274–289. 10.1007/s11336-016-9497-x 27052959 PMC4880644

[B31] McCrae R. R. John O. P. (1992). An introduction to the Five-Factor model and its applications. J. Pers. 60, 175–215. 10.1111/j.1467-6494.1992.tb00970.x 1635039

[B32] OECD (2013). Technical Report of the Survey of Adult Skills (PIAAC). Paris: OECD Publishing 10.1787/9789264204027-en

[B33] OECD (2015). The ABC of Gender Equality in Education: Aptitude, Behavior, Confidence. Paris: OECD Publishing 10.1787/9789264229945-en

[B34] Palmer S. P. Miles L. W. (2019). Students' observations of the nursing role in seven nations. Nurs. Educ. Perspect. 40, 283–290. 10.1097/01.NEP.0000000000000560 31436691

[B35] Putnick D. L. Bornstein M. H. (2016). Measurement invariance conventions and reporting: the state of the art and future directions for psychological research. Dev. Rev. 41, 71–90. 10.1016/j.dr.2016.06.004 27942093 PMC5145197

[B36] Rammstedt B. Beierlein C. (2014). Can't we make it any shorter? J. Ind. Diff. 35, 212–220. 10.1027/1614-0001/a000141

[B37] Rubin D. B. (1987). Multiple Imputation for Nonresponse in Surveys. New York, NY: Wiley 10.1002/9780470316696

[B38] Rust K. F. (1985). Variance estimation for complex estimators in sample surveys. J. Off. Stat. 1, 381–397.8931197

[B39] Rutkowski L. Gonzalez E. Joncas M. von Davier M (2010). International large-scale assessment data: issues in secondary analysis and reporting. Educ. Res. 39, 142–151. 10.3102/0013189X10363170

[B40] Rutkowski L. Gonzalez E. Von Davier M. Zhou Y. (2013). “Assessment design for international large-scale assessments,” in Handbook of International Large-Scale Assessment: Background, Technical Issues, and Methods of Data Analysis, eds L. Rutkowski, M. V. Davier, and D. Rutkowski (Boca Raton, FL: CRC Press), 75–95. 10.1201/b16061

[B41] Rutkowski L. Rutkowski D. (2018). Improving the comparability and local usefulness of international assessments: a look back and a way forward. Scand. J. Educ. Res. 62, 354–367. 10.1080/00313831.2016.1261044

[B42] Schleicher A. (2019). PISA 2018 Insights and Interpretations. Paris: OECD Publishing.

[B43] Schönberger K. Koberg T. (2018). Regional Data: Microcom. Bamberg: Research Data Center LIfBi.

[B44] Schultze M. Eid M. (2018). Identifying measurement invariant item sets in cross-cultural settings using an automated item selection procedure. Methodology 14, 177–188. 10.1027/1614-2241/a000155

[B45] Snyder P. Lawson S. (1993). Evaluating results using corrected and uncorrected effect size estimates. J. Exp. Educ. 61, 334–349. 10.1080/00220973.1993.10806594

[B46] Stankov L. Lee J. von Davier M. (2018). A note on construct validity of the anchoring method in PISA 2012. J. Psychoeduc. Assess. 36, 709–724. 10.1177/0734282917702270

[B47] Van de Vijver F. J. R. Avvisati F. Davidov E. Eid M. Fox J. P. Le Donné N.. (2019). “Invariance analyses in large-scale studies,” in OECD Education Working Papers (Paris: OECD Publishing).

[B48] von Davier M. (2013). “Imputing proficiency data under planned missingness in population models,” in Handbook of International Large-Scale Assessment: Background, Technical Issues, and Methods of Data Analysis, eds L. Rutkowski, M. V. Davier, and D. Rutkowski (Boca Raton, FL: CRC Press), 175–202.

[B49] von Davier M. Gonzalez E. Mislevy R. J. (2009). What are plausible values and why are they useful? IERI Monogr. Series 2, 9–36.

[B50] von Maurice J. Zinn S. Wolter I. (2017). Large-scale assessments: potentials and challenges in longitudinal designs. Psychol. Test Assess, Model. 59, 35–54.

[B51] Wagemaker H. (2014). “International Large-scale assessments: from research to policy,” in Handbook of International Large-Scale Assessment: Background, Technical Issues, and Methods of Data Analysis, eds L. Rutkowski, M. V. Davier, and D. Rutkowski (Boca Raton; London; New York, NY: CRC Press), 11–36.

[B52] Wohlkinger F. Ditton H. von Maurice J. Haugwitz M. Blossfeld H. P. (2011). 10 Motivational concepts and personality aspects across the life course. Zeitschrift Erziehungswissenschaft 14:155 10.1007/s11618-011-0184-5

